# Effect of Dentin Desensitizer Containing Novel Bioactive Glass on the Permeability of Dentin

**DOI:** 10.3390/ma15124041

**Published:** 2022-06-07

**Authors:** Ji-Hyun Jang, Hyun-Jung Kim, Joo-Young Choi, Hae-Won Kim, Samjin Choi, Soogeun Kim, Ayoung Bang, Duck-Su Kim

**Affiliations:** 1 Department of Conservative Dentistry, School of Dentistry, Kyung Hee University, Seoul 02453, Korea; jangjihyun@khu.ac.kr; 2 Department of Conservative Dentistry, Kyung Hee University Dental Hospital, Seoul 02453, Korea; kimhyunjung@khu.ac.kr; 3 Department of Conservative Dentistry, Graduate School, Kyung Hee University, Seoul 02453, Korea; choijooyoung@khu.ac.kr; 4 Institute of Tissue Regeneration Engineering (ITREN), Dankook University, Cheonan 31116, Korea; kimhw@dku.edu; 5 Department of Biomedical Engineering, College of Medicine, Kyung Hee University, Seoul 02453, Korea; medchoi@khu.ac.kr (S.C.); sigamda@khu.ac.kr (S.K.); ayoung.bang@gmail.com (A.B.)

**Keywords:** bioactive glass, dentin desensitizer, dentin hypersensitivity, real-time dentinal fluid flow, Raman spectroscopy

## Abstract

The objective of this study was to evaluate the effect of novel bioactive glass (BAG)-containing desensitizers on the permeability of dentin. Experimental dentin desensitizers containing 3 wt% BAG with or without acidic functional monomers (10-MDP or 4-META) were prepared. A commercial desensitizer, Seal & Protect (SNP), was used as a control. To evaluate the permeability of dentin, real-time dentinal fluid flow (DFF) rates were measured at four different time points (demineralized, immediately after desensitizer application, after two weeks in simulated body fluid (SBF), and post-ultrasonication). The DFF reduction rate (ΔDFF) was also calculated. The surface changes were analyzed using field emission scanning electron microscopy (FE-SEM). Raman spectroscopy was performed to analyze chemical changes on the dentin surface. The ΔDFF of the desensitizers containing BAG, BAG with 10-MDP, and BAG with 4-META significantly increased after two weeks of SBF storage and post-ultrasonication compared to the SNP at each time point (*p* < 0.05). Multiple precipitates were observed on the surfaces of the three BAG-containing desensitizers. Raman spectroscopy revealed hydroxyapatite (HAp) peaks on the dentin surfaces treated with the three BAG-containing desensitizers. Novel BAG-containing dentin desensitizers can reduce the DFF rate about 70.84 to 77.09% in the aspect of reduction of DFF through the HAp precipitations after two weeks of SBF storage.

## 1. Introduction

Dentin hypersensitivity (DH) is one of the most common clinical problems, and is characterized by short and sharp pain induced by thermal, tactile, evaporative, osmotic, or chemical stimuli of dentinal tubules causing fluid movement in accordance with the hydrodynamic theory [1]. The prevalence has been estimated to range from 4% to 74% of patients; it is especially prevalent in females, and its prevalence increases with age [1,2,3].

The management of DH can be conceptually categorized in two different strategies: nerve desensitization through suppression of the nerve excitation of A fibers using potassium ions, and mechanical occlusion of the dentinal tubules [3]. Most in-office treatments occlude or seal the dentinal tubules using various methods, including the use of dentin adhesive, protein coagulants such as glutaraldehyde or silver nitrate, the plugging of dentinal tubules using fluoride or oxalate, and laser treatment [3,4]. However, most of these treatments have shown a short-term maintenance effect, and thus repetitive applications are required along with a decrease in the treatment performance [4]. Resin-based adhesive systems comprising varnish, dentin adhesive, and resin-based desensitizers have exhibited long-term desensitizing effects in comparison to other topical agents owing to their good adhesion performance [5].

Bioactive glass (BAG) was first introduced as a bio-inert material for surgical implants to stimulate bone regeneration in tissue engineering. BAG can induce remineralization through ion exchange and by forming a hydroxycarbonate apatite (HCA) layer [6]. HCA is regarded as a precursor of HAp because they have similar chemical compositions, and it is considered to chemically interact with collagen fibrils and promote tissue mineralization [7]. It has been reported that BAG has anti-bacterial and anti-inflammatory effects [8] as well as good biocompatibility with dental pulp cells [9]. Thus, the supplementation of BAG into various dental restorative materials has been investigated. Dental composite resin or adhesive materials incorporating BAG have been reported to provide reduction of biofilm formation in pre-existing marginal gaps [8], remineralization of demineralized dentin [10,11,12], and prevention of demineralization and remineralization of enamel [13,14]. In addition, BAG showed ability to occlude dentinal tubule through the formation of HCA layer [15,16,17,18]. It has been applied in desensitizing toothpaste and prophylactic powders for the purpose of desensitization, and improved dentin permeability has been reported.

Although BAG containing desensitizing agents has been reported to be effective for DH, the duration of its effect was not prolonged owing to the application type of the agents, which were mostly slurry gels or pastes [15,16,17].

Contemporary dentin adhesives can be categorized as ‘etch-and-rinse’ or ‘self-etching’ approaches. The latter contains acidic functional monomers in place of a separate etching process; therefore, it has the advantage of a lower incidence of post-operative sensitivity owing to the omission of the ambiguity of wet-bonding, with favorable long-term clinical performance [19,20]. For self-etching approaches, various functional monomers have been suggested to produce a hybrid layer by dissolving the smear layer through their acidity and ionic interaction with hydroxyapatite (HAp), such as dipentaerythritol penta acrylate monophosphate (PENTA), 10-methacryloyloxydecyl dihydrogen phosphate (10-MDP), and 4-methacryloxyethyl trimellitic anhydride (4-META) [19]. Topical application of dentin adhesive as a desensitizer is also a well-known strategy to manage DH. The rationale of this method is to decrease the dentin permeability through mechanical occlusion of exposed dentinal tubules. A commercial self-etching dentin desensitizer, Seal & Protect (SNP), containing PENTA, has been reported to be effective in sealing dentin root surfaces for a prolonged duration [21].

Thus, we prepared experimental dentin desensitizers containing novel BAG to improve the sealing effect and extend the duration of desensitization. The aim of this study was to investigate the effect of novel BAG-containing dentin desensitizers on the dentin permeability through dentinal fluid flow (DFF) rate measurements, field emission scanning electron microscopy (FE-SEM), and Raman spectroscopy. Acidic functional monomers (either 10-MDP or 4-META) were also incorporated into the desensitizers to evaluate the additional effect of desensitizers. The following null hypotheses were evaluated: (1) there would be no difference in dentin permeability owing to the effect of BAG incorporation into the dentin desensitizer; and (2) there would be no differences in dentin permeability with the addition of acidic functional monomers to novel BAG-containing dentin desensitizers.

## 2. Materials and Methods

### 2.1. Specimen Preparation

A total of 40 extracted human premolars were obtained under a protocol approved by the Institutional Review Board of the Kyung Hee University Dental Hospital (KHD IRB 1811-3). The flat coronal dentin surface was exposed using a high-speed water-cooled diamond saw (Isomet 5000; Buehler Ltd., Lake Bluff, IL, USA). The surface was polished using 180-, 320-, and 600-grit silicon carbide (SIC) paper in ascending order to produce a standard smear layer. Next, 20 teeth were randomly assigned to the DFF rate measurements, another 16 were assigned to the FE-SEM analysis of the dentin surface, and the remaining 4 teeth were used for the Raman spectroscopy analysis.

### 2.2. BAG Preparation

BAG without amination was prepared via sol-gel synthesis according to the procedure reported by Lee et al. [22]. Briefly, a mixture of a precursor (calcium nitrate tetrahydrate), co-solvents (ethanol and 2-ethoxyethanol), surfactant (hexadecyltrimethylammonium bromide (CTAB)), and catalyst (aqueous ammonia) was prepared in deionized water (DW) at room temperature. After stirring for 30 min, tetraethyl orthosilicate was added at a Ca:Si molar ratio of 15:85. The mixture was stirred for 4 h until a gel formed. The precipitate was then filtered from the solution, washed, and dried for 24 h. Then, the precipitate was heated to remove CTAB. After calcination at 600 °C for 5 h, the precipitate was washed with ethanol and DW. Finally, BAG was obtained after drying under vacuum.

### 2.3. Experimental Groups

**Figure 1:**
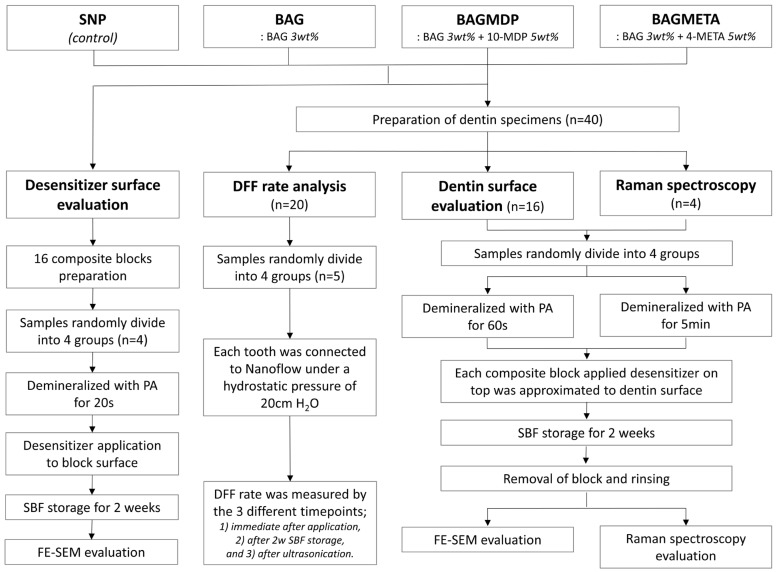
Classification of experimental groups and analysis methods.

Four experimental groups were assigned in this study. Commercial dentin desensitizer (Seal & Protect; Dentsply, Milford, DE, USA) was used as a control. We prepared three experimental dentin desensitizers containing BAG with or without acidic functional monomers, and the chemical compositions of the desensitizers used in this study are listed in Table 1. The concentrations of BAG and acidic functional monomers were determined according to a previously performed pilot study. Figure 1 shows the overall experimental procedures of this study.

### 2.4. Real-Time Dentinal Fluid Flow Reduction Rate (ΔDFF) Measurements

A total of 20 teeth randomly assigned to 4 experimental groups (n = 5) were used for the DFF rate measurements. The specimens were prepared as described in a previous study [12]. Briefly, the flat dentin surface was exposed, the root portion was removed 5 mm from the cemento-enamel junction, and the remaining pulp tissue was completely removed. Each specimen was fixed on an acryl plate with a hole, and a metal tube (0.9 mm in diameter) was inserted such that it connected to the pulp chamber, followed by sealing the exposed root surface using dentin adhesive (All-Bond Universal; Bisco) and a flowable composite resin (G-aenial Universal Flo; GC, Tokyo, Japan) to prevent unexpected leakage. The prepared specimen was stored in distilled water for 24 h, and then connected to a water reservoir with a hydrostatic pressure of 30 cm H_2_O to reproduce the physiologic pulpal pressure [23].

The prepared specimen was subsequently connected to a sub-nanoliter-scale DFF rate measuring device (NanoFlow-II; IB Systems, Seoul, Korea) and allowed to stabilize for 10 min before each measurement. The DFF rate measurements were performed in real-time, and data were acquired at four different time points: demineralized, immediately after desensitizer application, after two weeks of storage in SBF, and post-ultrasonication.

The ‘demineralized’ condition describes the specimens for which acid-etching of the dentin surface was performed for 60 s to remove the smear layer and open the dentinal tubules, followed by rinsing and blot-drying. In the ‘immediately after desensitizer application’ condition, the DFF rate was measured for 5 min throughout the application of each experimental desensitizer and light curing. Seal & Protect was applied according to the manufacturer’s instructions. Three experimental desensitizers were applied with gentle agitation and light cured for 20 s using an LED curing unit (Bluephase G2; Ivoclar Vivadent, Schaan, Liechtenstein) emitting 1200 mW/cm^2^. The specimens were stored in Tris simulated body fluid (SBF) [24,25] at 37 °C for two weeks (the solution was changed every two days to prevent autogenous precipitation), followed by measurement of the DFF rate for 5 min to obtain the ‘two week storage in SBF’ data. The chemical composition of the SBF solution is followed by Kim et al. [11]. Then, the specimens underwent ultrasonication for 3 min (Soniclean 160HT; Soniclean Pty Ltd., Thebarton, Australia), followed by measurement of the DFF rate for 5 min [26] to obtain the ‘post-ultrasonication’ data.

The reported DFF rates were calculated as the average of each measured real-time DFF rate for the four different time points. Due to the wide variation in the DFF rates for each tooth measured, the percentage change in the DFF reduction rate (ΔDFF) was calculated to compensate for the differences in the permeability of the teeth. The ΔDFF are defined as follows:

- ΔDFF_Immediate_ (%) = (DFF_Demineralized_ − DFF_Immediate_)/DFF_Demineralized_ × 100;
- ΔDFF_2w storage in SBF_ (%) = (DFF_Demineralized_ − DFF_2w in storage SBF_)/DFF_Demineralized_ × 100;
- ΔDFF_Post-ultrasonication_ (%) = (DFF_Demineralized_ − DFF_Post-ultrasonication_)/DFF_Demineralized_ × 100.

### 2.5. FE-SEM Analysis of the Desensitizer Surface

Sixteen composite blocks (Any-Com; MEDICLUS, Cheongju, Korea) were fabricated using a silicone mold (3.0 × 3.0 × 4.0 mm) and assigned to each group (n = 4). The top surface of the composite blocks was etched with 37% phosphoric acid gel (Etch-37; Bisco, Schaumburg, IL, USA) for 20 s and washed with water. The desensitizers were then applied to the surface of the blocks and light-cured for 20 s. Four groups of composite blocks were stored in SBF at 37 °C for two weeks, and the solution was changed every two days to prevent autogenous precipitation. After storage, the specimens were rinsed with distilled water for 3 min, completely dehydrated according to the procedure reported by Perdigao et al. [27], and sputter-coated with gold particles. Each block was then examined using FE-SEM (S-4700; Hitachi, Tokyo, Japan) at 10 kV.

### 2.6. FE-SEM Analysis of the Dentin Surface

Sixteen teeth were assigned to four experimental groups (n = 4). The flat dentin surface of each tooth was exposed and demineralized for 60 s with 37% phosphoric acid gel (Etch-37). Sixteen composite resin blocks with four experimental desensitizing agents on top of the composites were prepared as described in Section 2.5. The composite blocks were approximated to the demineralized dentin surface using orthodontic bands and stored in SBF for two weeks. After storage, the composite blocks were removed from the dentin surfaces, and half of the specimens in each group were ultrasonicated for 3 min. FE-SEM was performed for each dentin surface following the method described previously.

### 2.7. Raman Spectroscopy Analysis

To identify dentin remineralization, Raman spectroscopy (UniDRON, Yongin, Korea) was performed for all experimental groups using a 785 nm diode laser with 100 mW power and a 10× objective lens with 0.25 NA. Raman spectra were obtained at 25 random locations within the fingerprint range of 800–1100 cm^−1^ with a spectral resolution of 3 cm^−1^ and an acquisition time of 10 s. The characteristic peak of the hydroxyapatite (HAp) mineral is located at 960 cm^−1^ for the phosphate group [28], which is used as an indicator to evaluate changes in the mineral content of the specimen. The four teeth for Raman spectroscopy were divided into four groups. Raman spectra were measured at 5 × 5 mapping points with a total acquisition time of 5 min for the exposed dentin surface before demineralization and after acid-etching for 15 s [28]. The composite block with the applied agent was then attached to the dentin surface and stored in SBF as described above. After two weeks, the composite block was removed from the specimen, and Raman analysis was performed. Finally, the specimen was ultrasonicated for 3 min, and the Raman spectrum was measured again.

## 3. Statistical Analysis

Two-way analysis of variance (ANOVA) with a Dunnet test was used to determine the statistically significant differences in the ΔDFF of experimental groups at the 95% confidence level. Statistical analysis was performed using SPSS software (ver. 23.0.0; IBM Corp., Armonk, NY, USA). Differences were considered statistically significant at *p* < 0.05.

## 4. Results

### 4.1. Real-Time Dentinal Fluid Flow Reduction Rate (ΔDFF) Measurement

**Figure 2:**
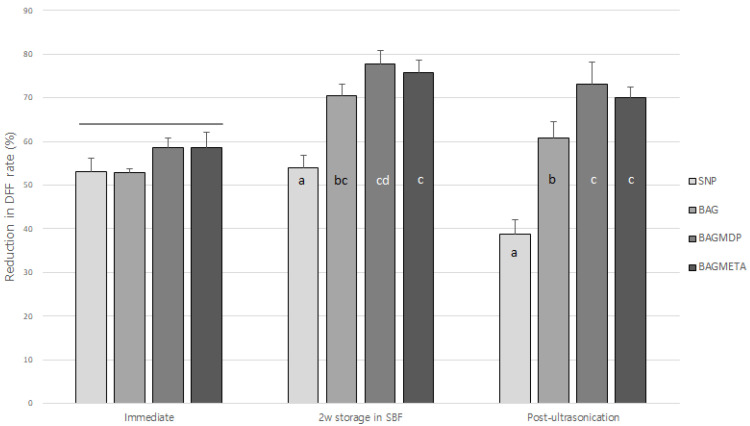
Changes in dentin fluid flow reduction rate of experimental groups at three different timepoints. Different letters indicate statistically significant differences between the materials at each timepoint. Long bar means no significant differences among groups.

**Figure 3:**
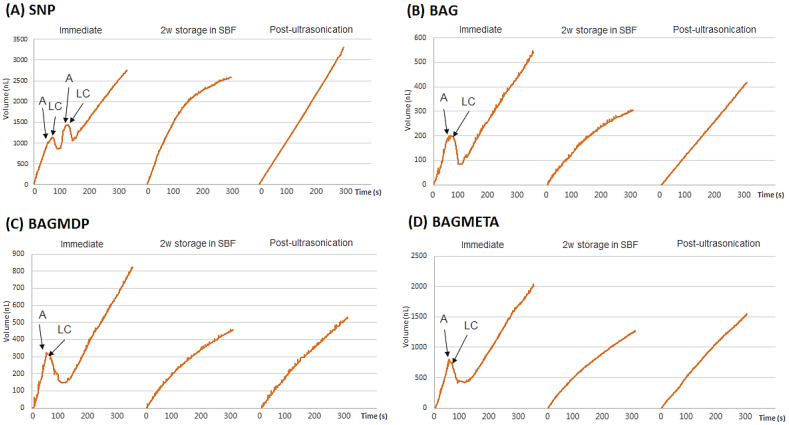
Representative graphs of real-time changes of DFF rate (%) of all experimental groups throughout the procedures, which consisted of the following three different timepoints: (1) before and after the application of desensitizer; (2) after the 2-week storage in SBF; (3) after ultrasonication. The DFF rate at each timepoint was measured for 5 min. The DFF rates (nL/s) are shown below the bar graphs, and the changes of DFF rate (%) are indicated with blue letters. Abbreviations: A, application of experimental desensitizer; LC, light-curing; SBF, simulated body fluid; SNP, Seal & Protect desensitizer; BAG, bioactive glass containing desensitizer; BAGMDP, bioactive glass and 10-Methacryloyloxydecyl dihydrogen phosphate containing desensitizer; BAGMETA, bioactive glass and 4-Methacryloxyethyltrimellitate anhydride.

Table 2 and Figure 2 present the DFF rates and ΔDFF for all experimental groups. For the immediate time points, there was no significant difference in ΔDFF_Immediate_ among the four groups (*p* > 0.05). After two weeks of storage in SBF, the ΔDFF_2w storage in SBF_ of all the experimental groups increased, except for the SNP group (*p* < 0.05). Ultrasonication decreased the ΔDFF_Post-ultrasonication_ of all the experimental groups compared to ΔDFF_2w storage in SBF_, except for the BAGMDP group (*p* < 0.05). Figure 3 shows representative real-time changes in the DFF rate of each group.

### 4.2. FE-SEM Analysis of the Desensitizer Surface

**Figure 4:**
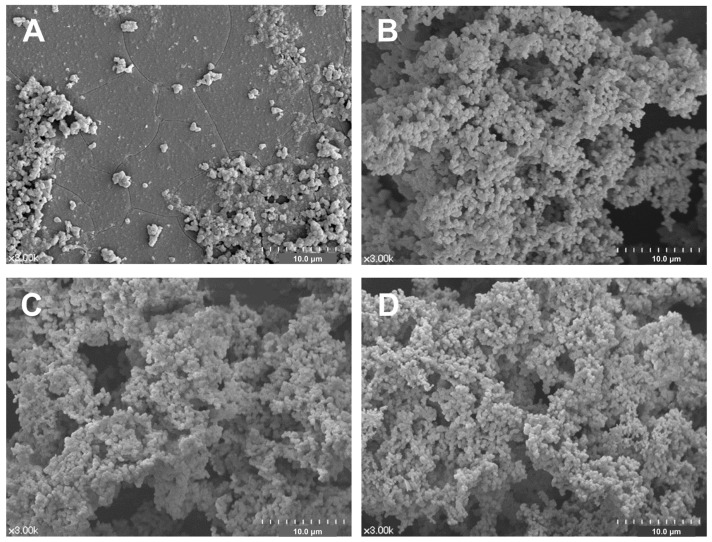
Representative surface morphology of the all experimental desensitizing agents after 2-week storage in SBF: (**A**) SNP, (**B**) BAG, (**C**) BAGMDP, and (**D**) BAGMETA groups.

Representative FE-SEM images of all experimental groups are shown in Figure 4. Abundant deposition of precipitates with multiple aggregates were observed on the surfaces of the BAG-containing groups (Figure 4B–D), whereas few precipitates were observed on the surface of the SNP group (Figure 4A).

### 4.3. FE-SEM Analysis of the Dentin Surface

**Figure 5:**
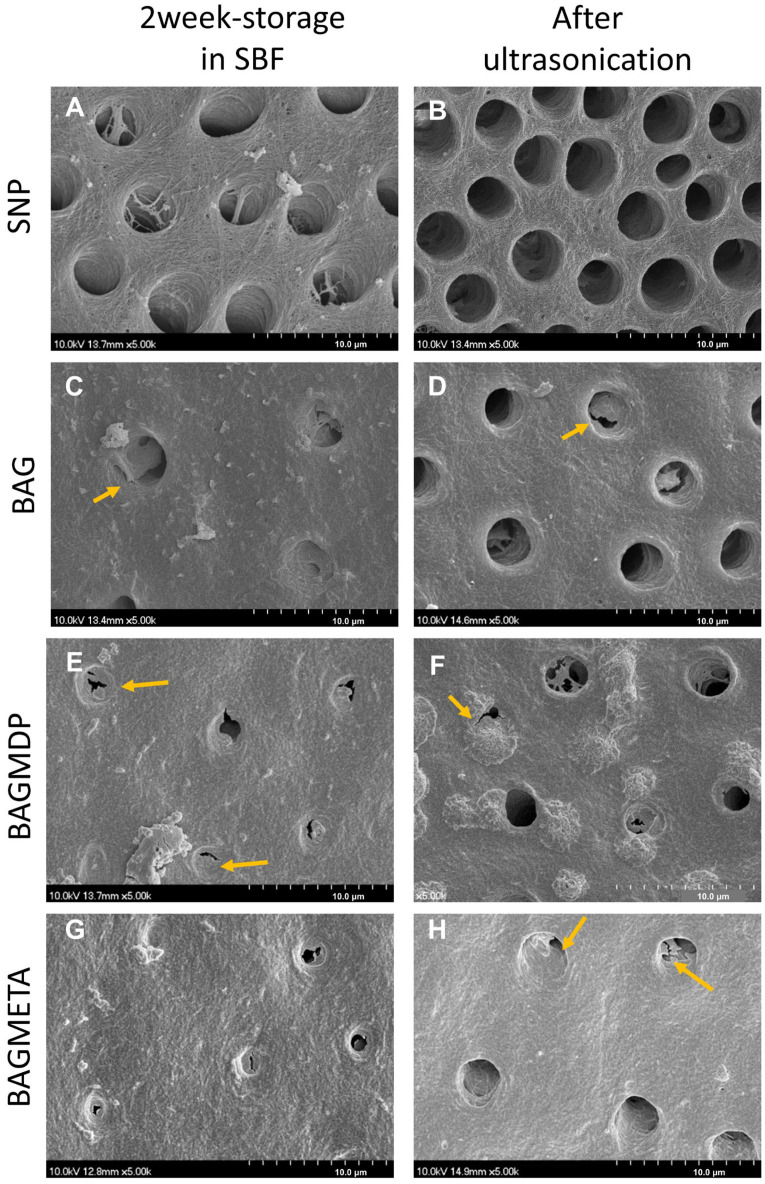
Representative SEM images of dentin surfaces: (**A,B**) SNP, (**C,D**) BAG, (**E,F**) BAGMDP, and (**G,H**) BAGMETA groups. SNP group (**A,B**). Left column images show the dentin surface after 2-week storage period in SBF, and right column images show the dentin surface after application of ultrasonication. The images present that the BAG-containing groups occluded exposed dentin surfaces with precipitates (arrows) and those occlusions were retained after the ultrasonication.

Representative FE-SEM images of each dentin surface to which the desensitizer was applied are shown in Figure 5. The partial occlusion of dentinal tubules was observed in the three BAG-containing groups (Figure 5C,E,G), whereas most dentinal tubules were left open in the SNP group (Figure 5A). The dentinal tubules in the SNP group were entirely opened after ultrasonication (Figure 5B). However, the occlusion of dentinal tubules was maintained in three BAG-containing groups (Figure 5D,F,H).

### 4.4. Raman Spectroscopy Analysis

**Figure 6:**
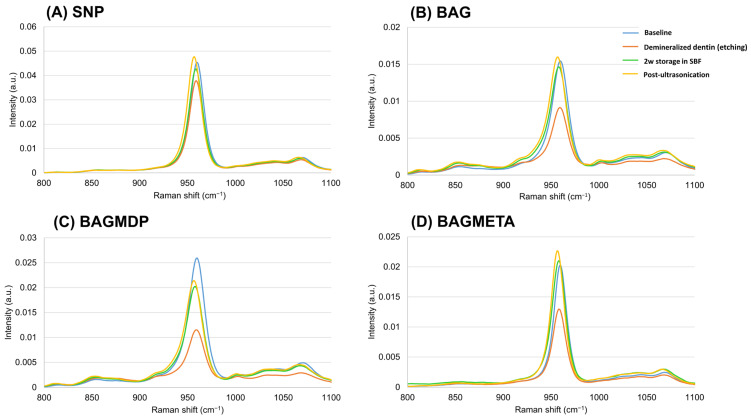
Representative graph of Raman spectra before and after demineralization, 2w storage in SBF of desensitizer-applied dentin, and after ultrasonication.

Figure 6 shows the Raman spectra for all the experimental groups. The most prominent peak for sound dentin in the range of 800 to 1100 cm^−1^ was found at 959 cm^−1^, which corresponds to the HAp phase of the dentin [29]. After demineralization, all groups showed a decrease in the peak at 959 cm^−1^. For the specimens after two weeks of storage in SBF, the intensity of the HAp peak increased by 60%, 75%, and 62% in the BAG, BAGMDP, and BAGMETA groups, respectively, compared with that after demineralization, whereas it increased by approximately 13% in the control group. After ultrasonication, the HAp peak was maintained or increased slightly in all groups.

## 5. Discussion

In the current study, the effect on dentin permeability of novel BAG-containing dentin desensitizers with or without acidic functional monomers of 10-MDP and 4-META was investigated. There was a significant increase in ΔDFF after two weeks of storage in SBF for the three BAG-containing desensitizers; thus, the first null hypothesis was rejected. The second null hypothesis was partially rejected because the ΔDFF_2w storage in SBF_ of three BAG-containing groups increased more than ΔDFF_Immediate_ and were also not significantly different from each other (*p* > 0.05); however, the ΔDFF_Post-ultrasonication_ of the BAGMDP groups remained significantly higher than those of the BAG and BAGMETA groups (*p* *<* 0.05).

As described in the introduction, BAG-containing desensitizing agents have been introduced. Among them, a representative commercial toothpaste is Novamin (NovaMin Technology Inc., Alachua, FL, USA). However, the particle size of NovaMin is approximately 18 μm (*D*_50_) [30]. Considering that the thickness of the cured layer of most dentin adhesives is approximately 10–20 μm, it is difficult to incorporate NovaMin into resin-based dentin desensitizers. However, the BAG used in this study has a particle size of 160 nm [22]. Thus, it can be incorporated into desensitizers without compromising the thickness of the cured desensitizer. To the best of our knowledge, BAG-containing light-curable dentin desensitizers have rarely been investigated for the management of DH.

The changes in dentin permeability were measured in real-time using the sub-nanoliter-scaled fluid flow measuring device, NanoFlow-II. This device has been used successfully in other studies requiring the accurate calculation of dentinal tubular fluid movement [31,32,33]. In the light-curing step, the DFF changes negatively because the tubular fluid moves toward the pulp as a result of thermal expansion [33]. Thereafter, the rebounding effect causes the DFF rate to increase rapidly and then to exhibit a constant flow rate. As shown in Figure 3, for the SNP group, two consecutive applications and light-curing procedures were performed according to the manufacturer’s instructions; the thin first layer of the SNP acts as a permeable membrane for the all-in-one adhesive, and the second layer is applied to enhance the sealing effect. In the three BAG-containing experimental groups, although the desensitizers were applied as single layers, the immediate ΔDFF was comparable to that of the SNP group. In addition, the ΔDFF of those was higher than SNP group after two weeks of storage. The short-term effect of BAG on dentin permeability was also verified by Kim et al. [12]. In their study, a BAG-containing dentin adhesive without an acidic functional monomer was used. Considering the results of the two studies, the decrease in the DFF rate might be attributed to the remineralization effect of the incorporated BAG.

In this study, the DFF rates and standard deviations of SNP and the BAGMETA group were very high. This is attributed to the anatomical difference of the teeth used in this study. The diameter of the dentinal tubule differs by various factors (e.g., depth, ages, physiological or pathological condition) [34,35,36]. ΔDFF_Immediate_ of all experimental groups were similar to each other. However, a distinct difference in ΔDFF was found after two weeks storage. Whereas ΔDFF of the SNP group did not change, that of three BAG-containing groups increased significantly (*p <* 0.05). It is speculated that BAG exposed on the surface of desensitizer could occlude dentinal tubules. FE-SEM analysis of the dentin surface also supported this result. Ultrasonication decreased ΔDFF of all experimental groups, and ΔDFF of the SNP group is the lowest (*p <* 0.05). It can be thought that the occlusion by Seal & Protect was susceptible to mechanical stress. Three BAG-containing groups showed higher ΔDFF than the SNP group after ultrasonication. The BAGMDP group exhibited the highest ΔDFF (*p* < 0.05). The BAG and BAGMETA groups also showed increased ΔDFF compared to the immediate condition, although it was reduced compared to that after two weeks of storage in SBF. These results suggest that the precipitate crystals that formed on the dentinal surface are stable under mechanical stress. The HCA layer and collagen fibrils are chemically bonded, which is known to form a strongly bonded interface [6,37]. This effect of BAG may increase the longevity of the desensitizer by enhancing the desensitizer–dentin bonds.

Our results suggest that the addition of acidic functional monomers can contribute to increase ΔDFF. Although the ΔDFF of three BAG-containing desensitizer groups were not statistically different after two weeks of storage, ultrasonication negatively affected the ΔDFF of the BAG group. The FE-SEM analysis also showed that the dentinal tubule occlusion in the BAGMDP and BAGMETA groups was more obvious than that of the BAG group after ultrasonication. 10-MDP has been identified to produce a durable hybrid layer through the formation of a self-assembled nano-layer with calcium in HAp at the adhesive interface [38,39], which can contribute to the longevity of the resin–dentin bond. In addition, 4-META has also been used for more than two decades owing to its favorable adhesion to the tooth structure [19,40,41]. PENTA, a functional monomer used in the Seal & Protect, is also known to form chemical bonds with calcium ions remaining in the dentin [42]. However, unlike the molecular structure of the 10-MDP and 4-META monomers, which have linear structures, PENTA has a high viscosity because of its three-dimensional spatial molecular structure with four additional vinyl groups, making it difficult to approach for chemical bonding [43]. This might be a reason why the control SNP group showed a substantial increase in the DFF rate after ultrasonication. In spite of discriminative results of DFF rate measurement, there are two limitations in this procedure. One was demineralization agent of dentin. Phosphoric acid was used in this study to make the baseline of real-time fluid movement measurement. However, it may not simulate the sensitive dentin requiring desensitization and the use of 6% citric acid would be better [44]. The other was the use of ultrasonication to evaluate stability of the desensitizer. Although it was effective in this study, the combination of erosive challenge and sonication is more proper for evaluation [45].

In the FE-SEM analysis of the desensitizer surface, the surface of the SNP was covered with small precipitates even though it did not contain BAG. However, the dentin surface of SNP was not occluded. Thus, these precipitates may be attributed to autogenous calcium phosphate precipitation in the SBF solution. Although we changed the SBF every two days to prevent autogenous precipitates, the prevention was not perfect. There were many precipitates on the surface of three BAG-containing desensitizers, and dentin surfaces were also occluded. Considering the two FE-SEM analyses, the precipitates formed on the surface of the BAG-containing desensitizers may be an HCA layer, which remineralized the dentin surface by reacting with the demineralized collagen fibers. Each surface of the desensitizer and desensitizer-treated dentin were analyzed separately with FE-SEM. The reason was that the interface of the desensitizer-dentin was likely to deform or destruct during specimen preparation. Although this method has been used in other studies [12,14,46] and is also valid in this study, it still lacks clinical relevance. Cross-sectional interface analysis with hydraulic conductance should be necessary in a future study.

We performed Raman spectroscopy for the same specimen at different time points to examine the chemical change in the dentin surface more accurately. The peak at 959 cm^−1^ is regarded as being most sensitive to mineral changes. When the three BAG-containing desensitizers were applied to demineralized dentin, this peak changed distinctly. Our findings are consistent to those of other studies [29,47]. Although this peak cannot represent complete remineralization histologically, it supports the results of the DFF reduction rate measurements and FE-SEM. Khalid et al. suggested measurement of the mineral-to-matrix ratio between 960 and 1650 cm^−1^, which can represent the volumetric fraction of mineral with respect to collagen [48]. Pezzoti et al. and Adachi et al. recommended using the full width at half maximum (FWHM) of the peak at 959 cm^−1^ to determine the degree of crystallinity and defects in the HA crystals [49,50]. Additional Raman spectral analyses such as the mineral to matrix ratio and Gaussian decomposition [51] will be necessary to evaluate the degree of dentin remineralization in further studies.

## 6. Conclusions

Within the limitations of this study, BAG-containing desensitizing agents were effective for reducing the DFF rate about 70.84 to 77.09% through precipitation of HAp crystals after 2 weeks of SBF storage. In addition, acidic functional monomers such as 10-MDP and 4-META did not hamper the remineralization ability of BAG, but enhanced the retention of the desensitizers, especially with 10-MDP (*p* < 0.05). It is suggested that a BAG-containing desensitizer with 10-MDP acidic monomer might be helpful to reduce DH and retain the desensitizing effect for a longer period.

## Figures and Tables

**Table 1:** Experimental groups and descriptions of the materials used in this study.

Group	Description of Experimental Material (Product, Manufacturer)	Chemical Composition
SNP	Commercial resin-based desensitizer (Seal & Protect, Dentsply Sirona, Tulsa, OK, USA)	Di-and tri-methacrylate resin, PENTA, functionalized amorphous silica, photoinitiator, butylated hydroxytoluene, cetylamine hydrofluoride, triclosan, acetone
BAG	BAG-containing desensitizer	85SBAG (3%), UDMA (42.8%), HEMA (12.2%), CQ (0.5%), ethanol (40%), BHT (0.25%), TP (0.25%), EDMAB (1%)
BAGMDP	Desensitizer containing BAG and 10-MDP	85SBAG (3%), 10-MDP (5%), UDMA (37.8%), HEMA (12.2%), CQ (0.5%), ethanol (40%), BHT (0.25%), TP (0.25%), EDMAB (1%)
BAGMETA	Desensitizer containing BAG and 4-META	85SBAG (3%), 4-META (5%), UDMA (37.8%), HEMA (12.2%), CQ (0.5%), ethanol (40%), BHT (0.25%), TP (0.25%), EDMAB (1%)

Abbreviations: PENTA, dipentaerythritol penta acrylate monophosphate; UDMA, urethane dimethacrylate; HEMA, hydroxyl ethylene glycolmethacrylate; CQ, camphorquinone; BHT, 2,6-di-tert-butyl-4-hydroxytoluene; TP, 2,2′-(P-Tolylimino)-diethanol; EDMAB, ethyl-4-dimethylaminobenzoate; 10-MDP, methacryloyloxydecyl dihydrogen phosphate; 4-META, 4-methacryloxyethyltrimellitate anhydride. ‘%’ means weight %.

**Table 2:** Real-time changes in DFF rate and ΔDFF (n = 5).

		SNP	BAG	BAGMDP	BAGMETA
DFF rate (nL/s)	Demineralized	6.50 ± 6.77	3.68 ± 1.02	4.62 ± 2.40	7.30 ± 6.00
	Immediate	3.04 ± 3.38	1.74 ± 0.54	1.91 ± 0.95	3.02 ± 2.27
	2w storage in SBF	2.99 ± 3.17	1.09 ± 0.36	1.03 ± 0.53	1.78 ± 1.41
	Post-ultrasonication	3.98 ± 4.28	1.45 ± 0.46	1.25 ± 0.64	2.18 ± 1.71
ΔDFF (%)	ΔDFF_Immediate_	54.63 ± 3.19 ^aB^	53.35 ± 3.10 ^aA^	57.69 ± 3.84 ^aA^	57.13 ± 3.71 ^aA^
	ΔDFF_2w in SBF_	54.29 ± 1.04 ^aB^	70.84 ± 2.93 ^bcC^	77.09 ± 4.31 ^cdB^	75.18 ± 3.26 ^cC^
	ΔDFF_Post-ultrasonication_	39.44 ± 2.34 ^aA^	61.12 ± 3.35 ^bB^	72.39 ± 5.74 ^cB^	69.61 ± 2.70 ^cB^

Values are written as mean ± standard deviation. Within the same column and row, mean values with different capital letters and superscript lowercase letters represent significant differences, respectively (*p* < 0.05). Abbreviations: SNP, Seal & Protect desensitizer; BAG, desensitizer containing bioactive glass; BAGMDP, desensitizer containing bioactive glass and 10-methacryloyloxydecyl dihydrogen phosphate; BAGMETA, desensitizer containing bioactive glass and 4-methacryloxyethyltrimellitate anhydride; SBF, simulated body fluid.

## Data Availability

The data presented in this study are available on request from the corresponding author.
